# Associations between cord blood metabolic factors and early-childhood growth and overweight and obesity

**DOI:** 10.3389/fendo.2023.1164747

**Published:** 2023-07-11

**Authors:** Wen-Yuan Jin, Xiao-Yang Chen, Ting Han, Yan Jin, Ting-Ting Chen, Zi-Han Wang, Zheng-Yan Zhao, Zhi-Wei Zhu

**Affiliations:** ^1^ Department of Developmental Behavioral Pediatrics, Children’s Hospital, Zhejiang University School of Medicine, National Clinical Research Center for Child Health, Hangzhou, China; ^2^ Department of Genetic and Metabolism, Children’s Hospital, Zhejiang University School of Medicine, National Clinical Research Center for Child Health, Hangzhou, China

**Keywords:** cord blood, metabolic factors, TG, HbA1c, C-peptide, growth, overweight and obesity

## Abstract

**Objective:**

This prospective cohort study was aimed at investigating the associations between cord blood metabolic factors and early-childhood growth, further elucidating the relationships between cord blood metabolites and overweight and obesity in early life.

**Methods:**

A total of 2,267 pairs of mothers and offspring were recruited in our study. Cord blood plasma was assayed for triglycerides (TGs), total cholesterol (TC), low-density lipoprotein-cholesterol (LDL-C), high-density lipoprotein-cholesterol (HDL-C), C-peptide, insulin, and glycosylated hemoglobin type A1C (HbA1c) levels. Data of anthropometric measurements were collected from offspring at birth, 6 months, 12 months, and 18 months. Multiple linear regression models were used to evaluate the correlations between cord blood metabolic factors and weight Z-scores, body mass index (BMI) Z-scores, and weight gains at the early stage of life. Forward stepwise logistic regression analyses were applied to explore the associations between cord blood metabolic factors and early-childhood overweight and obesity. Receiver operating characteristic (ROC) curve analyses were applied to determine the optimal cutoff points for cord blood metabolic factors in predicting early-childhood overweight and obesity.

**Results:**

After adjustments for covariates, cord blood TG concentrations and TG/TC ratios were negatively associated with weight Z-scores from birth to 18 months. Cord blood C-peptide and HbA1c levels were inversely associated with weight Z-scores at 6 months and 18 months. Cord blood TG concentrations and TG/TC ratios were negatively correlated with BMI Z-scores up to 18 months. Cord blood C-peptide levels and HbA1c levels were inversely correlated with BMI Z-scores at 18 months. Cord blood TG, TG/TC ratios, C-peptide, and HbA1c had negative correlations with weight gains from birth to 6 months, but the correlations attenuated as time went on. Increase in cord blood TG and HbA1c levels and TG/TC ratios were significantly associated with decreased risks of overweight and obesity at 6 months, 12 months, and 18 months.

**Conclusions:**

Cord blood metabolic factors were significantly associated with early-childhood growth patterns.

## Introduction

Evidence has suggested that maternal nutrition and the intrauterine metabolic environment play an important role in placental-fetal growth and development. According to the Developmental Origins of Health and Disease (DOHaD) theory, individuals exposed to an undernourished environment *in utero* were more prone to developing type 2 diabetes, obesity, or cardiovascular diseases in adulthood life as a result of epigenetic modifications (1, 2). Recent studies have implied that maternal glucose and lipid metabolic dysfunctions, including gestational diabetes mellitus (GDM) and hyperlipidemia, were closely related with childhood overweight and adiposity (3–5).

Umbilical cord blood metabolite levels were essential indicators reflecting intrauterine metabolic status of fetuses. It has been discovered that cord blood cardiometabolic factors were associated with fetal growth and birth weight status. Fetal weight was positively associated with insulin levels especially during the period of late gestation. Fetal weight was negatively correlated with triglyceride (TG) levels and positively correlated with high-density lipoprotein-cholesterol (HDL-C) levels during the third trimester of pregnancy (6). Results from prospective cohort studies showed that high cord serum TG levels and insulin levels were significantly associated with small for gestational age (SGA) and large for gestational age (LGA) neonates, respectively (7, 8). SGA newborns were more likely to have lower levels of total cholesterol (TC), low-density lipoprotein-cholesterol (LDL-C) and HDL-C in cord blood. Cord blood insulin levels were linearly associated with increased birth weights (8). Whereas cord blood TG levels were negatively associated with neonatal birth weights (9, 10). In addition, cord serum TG concentrations were found to have a strong negative association with insulin-like growth factor I (IGF-I), which assisted in predicting intrauterine growth restriction newborns (11). Umbilical cord leptin, known as a hormone regulating lipid metabolism through a negative feedback mechanism, was found to be positively correlated with neonatal birth weight and body mass index (BMI) (12).

Epidemiological studies further indicated that cord blood metabolic factors were associated with early-childhood growth and fat mass. A German research group followed up offspring’s postnatal growth trajectories (13, 14). Interestingly, they discovered that cord plasma insulin concentrations were positively correlated with neonatal birth weight and fat mass but inversely correlated with body weight gain up to 2 years old (13). Additionally, cord blood insulin was negatively associated with skinfold thicknesses and body fat percentage at 3 years old. The inverse relationship could also be observed between cord blood insulin and weight gain up to 5 years old in girls, but the effect size was small without clinical significance (14). In another prospective cohort study, cord blood insulin concentrations were found to be positively associated with skinfold thickness. Ghrelin concentrations were negatively associated with BMI and skinfold thickness at age 1 year (15). A meta-analysis retrieved 22 studies and concluded that umbilical cord blood leptin was positively associated with adiposity at birth but inversely related to adiposity up to 3 years of age. However, the association was not sustained up to 5 years old (16).

This multicenter prospective cohort study was aimed at investigating the associations between cord blood lipid and glucose metabolism and early-childhood growth, further elucidating the relationships between cord blood metabolic factors and overweight and obesity in early life.

## Materials and methods

### Study population

Between 1 January 2010 and 30 June 2011, pregnant women who attended regular prenatal examinations and intended to give birth in Yongkang, Huzhou, Cixi, Ninghai, Shaoxing, Yiwu, Dongyang, and Wenling Maternal and Child Health Care Hospitals were invited to participate in this study. Before recruitment, every participant signed the written informed consent. At the same time, our study obtained approval from the Clinical Research Ethics Committee of the hospitals. Inclusion criteria of pregnant women were 28–37 weeks of gestation, singleton pregnancy, naturally conceived, having clear gestational age, and integrated medical records. Exclusion criteria of mothers were multiple pregnancies, having type 1 or type 2 diabetes mellitus, chromosomal abnormalities, inherited metabolic diseases or thyroid diseases, being conceived with assisted reproductive technology, and experiencing severe infections during early pregnancy. Information about maternal age, educational background, family income, parity, prepregnancy BMI, cigarette exposure, pregnancy complications [e.g., GDM, hypertensive disorders in pregnancy, intrahepatic cholestasis of pregnancy (ICP), etc.] was sourced from questionnaire and medical records. They were followed up until delivery, and information regarding delivery mode, gestational age, infant gender, birth weight, birth length, Apgar scores, and perinatal complications was recorded. Inclusion criteria of neonates were singleton and postpartum 5-min Apgar scores ≥7. Exclusion criteria for neonates were congenital abnormalities, inherited metabolic diseases, and postpartum 5-min Apgar scores <7. Finally, a total of 2,267 pairs of mothers and offspring were recruited in our study. Among the subjects, 1,250 participants were boys and 1,017 participants were girls.

### Biochemical analyses

Venous umbilical cord blood samples were collected at birth in anticoagulant ethylenediaminetetraacetic acid (EDTA) tubes. Serum samples were obtained through centrifugation (3,500 rpm at 4°C for 10 min) and stored at -80°C until analysis. Every cord blood plasma was assayed for TG, TC, LDL-C, HDL-C, C-peptide, insulin, and glycosylated hemoglobin type A1C (HbA1c) levels on an automatic biochemical analyzer (Roche Cobase C8000, Roche Diagnostics, Germany). All of the inter- and intra-assay coefficients of variation were <2.0%.

### Anthropometric measurements

Offspring in our study were followed up and received regular physical examinations at birth, 6 months, 12 months, and 18 months. All of the data of anthropometric measurements were collected from standardized measuring devices. Weights were measured to the nearest 0.01 kg with an electronic scale (376, Hangzhou Seca Medical Measuring Apparatus Company, China). Lengths were measured to the nearest 0.1 cm with a length-measuring instrument (416, Hangzhou Seca Medical Measuring Apparatus Company, China). Both weight and length measurements were performed in light clothing. Z-scores of weight-for-age and BMI-for-age were calculated on the basis of WHO Child Growth Standards (17). Z-scores were calculated through the formula: Z-score = (x-μ)/σ.

### Definitions

BMI was calculated via the formula: BMI = weight/(height^2^). Maternal prepregnancy BMIs were categorized into underweight (<18.5 kg/m^2^), normal weight (18.5–24.9 kg/m^2^), overweight (25.0–29.9 kg/m^2^), and obesity (≥30.0 kg/m^2^) groups according to the WHO BMI classifications. Neonates were classified into appropriate/small/large for gestational age (AGA/SGA/LGA) based on Chinese Neonatal Birth Weight Curve for Different Gestational Age (18). Neonates were defined as AGA if their birth weights met or exceeded the 10th percentile and met or fell below the 90th percentile for gestational age. SGA/LGA were respectively defined as birth weights that fell below the 10th percentile/exceeded the 90th percentile for gestational age. Offspring overweight was defined as a BMI ≥85 percentile and <95 percentile among the same age and same gender children. Obesity was defined as a BMI ≥95 percentile among the same age and same gender children. Their reference values were based on the WHO Child Growth Standards (17).

### Statistical analysis

In the present study, categorical variables were presented as N (%). Normally distributed and skewed distributed continuous variables were presented as mean (standard deviation, SD) and median (interquartile range, IQR), respectively. Multiple linear regression models were used to examine the correlations between cord serum lipid profile, glucose metabolism, and growth at early stage of life. Forward stepwise logistic regression analyses were applied to explore the associations between cord blood metabolic factors and offspring overweight and obesity. ROC curve analyses were applied to determine the optimal cutoff points for cord blood metabolic factors in predicting early-childhood overweight and obesity. Each optimal cutoff point was obtained through searching for the maximum value of sensitivity+specificity–1 (Youden index). All of the data analyses were performed through SPSS 25.0 version for Windows (SPSS Inc., Chicago, USA). P values <0.05 were regarded as statistically significant.

## Results

### Characteristics of the study population

**Table 1** presented the basic characteristics of our study population. Among the expectant mothers recruited in the study, the mean (SD) age at delivery was 26.56 (3.80) years old. Most (72.0%) of them had a normal prepregnancy BMI, and only 4.9% of them were stratified as overweight or obese before pregnancy. The prevalence of GDM, hypertensive disorders in pregnancy, and ICP was respectively 10.2%, 3.0%, and 0.5%. Most (87.2%) of them were nulliparous. Nearly half (41.5%) of them were exposed to cigarette during pregnancy. Among the newborns, approximately 55% were boys. The mean (SD) gestational age at birth was 39.06 (1.17) weeks. About 2.0% of the neonates were preterm birth. Their mean (SD) birth weight was 3,367.8 (398.4) g. In addition, 78.8% of them were AGA, 5.8% of them were SGA, and 15.4% of them were LGA.

**Table 1:** Characteristics of the study population.

Characteristics	Mean ± SD or Median (IQR) or N (%)
Maternal characteristics	
Maternal age at delivery (years)	26.56 ± 3.80
Prepregnancy BMI (kg/m^2^)	20.38 ± 2.46
Underweight (<18.5)	501 (22.1%)
Normal weight (18.5–24.9)	1,632 (72.0%)
Overweight and obese (≥25.0)	111 (4.9%)
Unknown	23 (1.0%)
Maternal education	
≥University	411 (18.1%)
Junior college	804 (35.5%)
High school	394 (17.4%)
≤Junior school	648 (28.6%)
Unknown	10 (0.4%)
Family income (yuan/year)	
>200,000	118 (5.2%)
100,000–200,000	366 (16.1%)
50,000–100,000	898 (39.6%)
<50,000	872 (38.5%)
Unknown	13 (0.6%)
GDM	232 (10.2%)
Hypertensive disorders in pregnancy	67 (3.0%)
ICP	12 (0.5%)
Parity	
Nulliparous	1,977 (87.2%)
Multiparous	290 (12.8%)
Neonatal characteristics	
Male infant	1,250 (55.1%)
GA at delivery (week)	39.06 ± 1.17
Preterm (GA <37)	46 (2.0%)
Full term (37 ≤ GA < 42)	2,217 (97.8%)
Post term (GA ≥42)	4 (0.2%)
Birth weight (g)	3,367.8 ± 398.4
Weight for gestational age	
SGA	132 (5.8%)
AGA	1,787 (78.8%)
LGA	348 (15.4%)
Cord blood metabolic factors levels	
TC, mmol/L	1.66 (1.43-1.93)
TG, mmol/L	0.30 (0.23-0.38)
LDL-C, mmol/L	0.69 (0.56-0.84)
HDL-C, mmol/L	0.81 (0.67-0.98)
TG/TC	0.18 (0.14-0.23)
HDL/LDL	1.16 (0.95-1.44)
Insulin, μIU/ml	4.22 (2.15-7.18)
C-peptide, nmol/L	0.30 (0.21-0.42)
HbA1c, %	2.90 (2.60-3.10)

SD, standard deviation; IQR, interquartile range; BMI, body mass index; GDM, gestational diabetes mellitus; ICP, intrahepatic cholestasis of pregnancy; GA, gestational age; SGA, small for gestational age; AGA, appropriate for gestational age; LGA, large for gestational age; TC, total cholesterol; TG, triglyceride; LDL-C, low-density lipoprotein-cholesterol; HDL-C, high-density lipoprotein-cholesterol; HbA1c, glycosylated hemoglobin type A1C.

### Associations between cord blood metabolic factors and early-childhood weight Z-scores

Consistent with previous studies, we found that cord blood TG concentrations were strongly negatively associated with birth weights even when adjusted for maternal age, prepregnancy BMI, maternal education level, family income, parity, pregnancy complications, and child sex [β = -0.10; 95% confidence interval (CI) = -0.13, -0.07; P < 0.001]. Cord blood insulin concentrations had a positive association with birth weights when adjusted for confounding variables (β = 0.011; 95% CI = 0.008, 0.013; P < 0.001).

**Table 2** displayed the associations between cord blood metabolic factor levels and early-childhood weight Z-scores in our participants. After adjustments for maternal age, prepregnancy BMI, maternal education level, family income, parity, GDM, hypertensive disorders in pregnancy, ICP, child sex and birth weight, cord blood TG concentrations were found to be negatively associated with weight Z-scores at 6 months (β = -0.22; 95% CI = -0.37, -0.07; P = 0.004), 12 months (β = -0.19; 95% CI = -0.34, -0.05; P = 0.009), and 18 months (β = -0.19; 95% CI = -0.32, -0.06; P = 0.005). Cord blood TG/TC ratios were significantly negatively associated with weight Z-scores at 6 months (β = -0.62; 95% CI = -1.05, -0.19; P = 0.005), 12 months (β = -0.76; 95% CI = -1.17, -0.35; P < 0.001), and 18 months (β = -0.55; 95% CI = -0.93, -0.17; P = 0.004). Cord plasma C-peptide levels were inversely associated with weight Z-scores at 6 months (β = -0.26; 95% CI = -0.46, -0.07; P = 0.008) and 18 months (β = -0.26; 95% CI = -0.43, -0.08; P = 0.004). Cord plasma HbA1c levels were inversely associated with weight Z-scores at 6 months (β = -0.10; 95% CI = -0.17, -0.03; P = 0.005) and 18 months (β = -0.07; 95% CI = -0.13, -0.01; P = 0.022). Additionally, insulin concentrations had a negative association with 18-month weight Z-scores, but the relationship was very small without clinical significance.

**Table 2:** Associations between cord blood metabolic factors and early-childhood weight Z-scores.

Cord blood metabolic factors	6 months		12 months		18 months	
	Beta (95% CI)	P	Beta (95% CI)	P	Beta (95% CI)	P
TC	-0.06 (-0.13, 0.02)	0.124	0.02 (-0.05, 0.09)	0.586	0.01 (-0.06, 0.07)	0.843
TG	-0.22 (-0.37, -0.07)	0.004	-0.19 (-0.34, -0.05)	0.009	-0.19 (-0.32, -0.06)	0.005
LDL-C	-0.08 (-0.20, 0.04)	0.196	0.06 (-0.05, 0.18)	0.285	0.02 (-0.09, 0.13)	0.714
HDL-C	-0.03 (-0.18, 0.13)	0.748	0.08 (-0.07, 0.23)	0.290	0.09 (-0.05, 0.23)	0.189
TG/TC	-0.62 (-1.05, -0.19)	0.005	-0.76 (-1.17, -0.35)	0.000	-0.55 (-0.93, -0.17)	0.004
HDL/LDL	0.02 (-0.06, 0.10)	0.571	-0.04 (-0.11, 0.04)	0.358	-0.01 (-0.08, 0.07)	0.893
Insulin	-0.01 (-0.01, 0.00)	0.093	0.00 (-0.01, 0.00)	0.277	-0.01 (-0.01, 0.00)	0.027
C-peptide	-0.26 (-0.46, -0.07)	0.008	-0.17 (-0.36, 0.01)	0.070	-0.26 (-0.43, -0.08)	0.004
HbA1c	-0.10 (-0.17, -0.03)	0.005	-0.05 (-0.12, 0.01)	0.119	-0.07 (-0.13, -0.01)	0.022

Multiple linear regression was applied to examine the correlations between cord blood metabolic factors and early-childhood weight Z-scores. Z-score = (x-μ)/σ. Model was adjusted for maternal age, prepregnancy body mass index, maternal education level, family income, parity, gestational diabetes mellitus, hypertensive disorders in pregnancy, intrahepatic cholestasis of pregnancy, child sex and birth weight.

CI, confidence interval; TC, total cholesterol; TG, triglyceride; LDL-C, low-density lipoprotein-cholesterol; HDL-C, high-density lipoprotein-cholesterol; HbA1c, glycosylated hemoglobin type A1C.

### Associations between cord blood metabolic factors and early-childhood BMI Z-scores

**Table 3** showed the associations between cord blood metabolic factors levels and early-childhood BMI Z-scores in the population. After adjustments for the above covariates, cord blood TG concentrations were negatively correlated with BMI Z-scores at 6 months (β = -0.28; 95% CI = -0.45, -0.11; P = 0.001), 12 months (β = -0.28; 95% CI = -0.42, -0.13; P < 0.001), and 18 months (β = -0.23; 95% CI = -0.37, -0.08; P = 0.002). Cord blood TG/TC ratios were significantly negatively associated with BMI Z-scores at 6 months (β = -0.79; 95% CI = -1.27, -0.30; P = 0.001), 12 months (β = -1.03; 95% CI = -1.44, -0.62; P < 0.001), and 18 months (β = -0.57; 95% CI = -0.98, -0.16; P = 0.006). Cord plasma C-peptide levels (β = -0.19; 95% CI = -0.38, -0.01; P = 0.043) and HbA1c levels (β = -0.09; 95% CI = -0.15, -0.02; P = 0.009) were inversely correlated with BMI Z-scores at 18 months. In addition, cord plasma TC (β = -0.08; 95% CI = -0.16, 0.00; P = 0.047) and LDL-C (β = -0.14; 95% CI = -0.27, 0.00; P = 0.045) concentrations were inversely correlated with BMI Z-scores at 6 months, but the correlations attenuated at 12 months and 18 months.

**Table 3:** Associations between cord blood metabolic factors and early-childhood body mass index Z-scores.

Cord blood metabolic factors	6 months		12 months		18 months	
	Beta (95% CI)	P	Beta (95% CI)	P	Beta (95% CI)	P
TC	-0.08 (-0.16, 0.00)	0.047	-0.02 (-0.08, 0.05)	0.670	-0.03 (-0.09, 0.04)	0.453
TG	-0.28 (-0.45, -0.11)	0.001	-0.28 (-0.42, -0.13)	0.000	-0.23 (-0.37, -0.08)	0.002
LDL-C	-0.14 (-0.27, 0.00)	0.045	0.00 (-0.11, 0.12)	0.978	-0.06 (-0.17, 0.06)	0.340
HDL-C	-0.02 (-0.20, 0.16)	0.825	0.06 (-0.09, 0.21)	0.414	0.08 (-0.07, 0.23)	0.286
TG/TC	-0.79 (-1.27, -0.30)	0.001	-1.03 (-1.44, -0.62)	0.000	-0.57 (-0.98, -0.16)	0.006
HDL/LDL	0.07 (-0.02, 0.16)	0.117	-0.01 (-0.08, 0.07)	0.902	0.03 (-0.05, 0.10)	0.494
Insulin	0.00 (-0.01, 0.01)	0.801	0.00 (0.00, 0.01)	0.642	0.00 (-0.01, 0.00)	0.183
C-peptide	-0.07 (-0.29, 0.15)	0.533	-0.02 (-0.20, 0.17)	0.860	-0.19 (-0.38, -0.01)	0.043
HbA1c	-0.08 (-0.15, 0.00)	0.054	-0.05 (-0.12, 0.01)	0.125	-0.09 (-0.15, -0.02)	0.009

Multiple linear regression was applied to examine the correlations between cord blood metabolic factors and early-childhood BMI Z-scores. Z-score = (x-μ)/σ. Model was adjusted for maternal age, prepregnancy body mass index, maternal education level, family income, parity, gestational diabetes mellitus, hypertensive disorders in pregnancy, intrahepatic cholestasis of pregnancy, child sex and birth weight.

CI, confidence interval; TC, total cholesterol; TG, triglyceride; LDL-C, low-density lipoprotein-cholesterol; HDL-C, high-density lipoprotein-cholesterol; HbA1c, glycosylated hemoglobin type A1C.

### Associations between cord blood metabolic factors and early-childhood weight gains

**Table 4** exhibited the associations between cord blood metabolic factor levels and early-childhood weight gains in our participants. After adjustments for the covariates, cord serum metabolic factors were negatively correlated with weight gains from birth to 6 months, but the correlations attenuated as time went on. Cord serum TG (β = -0.20; 95% CI = -0.33, -0.07; P = 0.004), TG/TC ratios (β = -0.56; 95% CI = -0.94, -0.17; P = 0.005), C-peptide (β = -0.24; 95% CI = -0.41, -0.06; P = 0.008), and HbA1c (β = -0.09; 95% CI = -0.15, -0.03; P = 0.005) were negatively correlated with weight gains from birth to 6 months. Cord serum C-peptide had a weak inverse correlation with weight gains from 12 months to 18 months (β = -0.14; 95% CI = -0.28, 0.00; P = 0.044). However, cord serum TC concentrations (β = 0.07; 95% CI = 0.02, 0.12; P = 0.008) and LDL-C concentrations (β = 0.14; 95% CI = 0.05, 0.23; P = 0.002) were found to be positively associated with weight gains from 6 months to 12 months.

**Table 4:** Associations between cord blood metabolic factors and early-childhood weight gains.

Cord blood metabolic factors	0-6 months		6-12 months		12-18 months	
	Beta (95% CI)	P	Beta (95% CI)	P	Beta (95% CI)	P
TC	-0.05 (-0.11, 0.01)	0.124	0.07 (0.02, 0.12)	0.008	-0.01 (-0.06, 0.04)	0.626
TG	-0.20 (-0.33, -0.07)	0.004	-0.01 (-0.12, 0.10)	0.841	-0.04 (-0.15, 0.07)	0.484
LDL-C	-0.07 (-0.18, 0.04)	0.194	0.14 (0.05, 0.23)	0.002	-0.04 (-0.13, 0.04)	0.328
HDL-C	-0.02 (-0.16, 0.12)	0.748	0.11 (0.00, 0.23)	0.058	0.03 (-0.08, 0.14)	0.581
TG/TC	-0.56 (-0.94, -0.17)	0.005	-0.28 (-0.60, 0.04)	0.083	0.12 (-0.19, 0.43)	0.444
HDL/LDL	0.02 (-0.05, 0.09)	0.571	-0.06 (-0.12, 0.00)	0.048	0.03 (-0.02, 0.09)	0.252
Insulin	-0.01 (-0.01, 0.00)	0.093	0.00 (0.00, 0.01)	0.626	0.00 (-0.01, 0.00)	0.051
C-peptide	-0.24 (-0.41, -0.06)	0.008	0.05 (-0.10, 0.19)	0.536	-0.14 (-0.28, 0.00)	0.044
HbA1c	-0.09 (-0.15, -0.03)	0.005	0.03 (-0.02, 0.08)	0.237	-0.03 (-0.08, 0.01)	0.169

Multiple linear regression was applied to examine the correlations between cord blood metabolic factors and early-childhood weight gains. Model was adjusted for maternal age, prepregnancy body mass index, maternal education level, family income, parity, gestational diabetes mellitus, hypertensive disorders in pregnancy, intrahepatic cholestasis of pregnancy, child sex and birth weight.

CI, confidence interval; TC, total cholesterol; TG, triglyceride; LDL-C, low-density lipoprotein-cholesterol; HDL-C, high-density lipoprotein-cholesterol; HbA1c, glycosylated hemoglobin type A1C.

### Associations between cord blood metabolic factors and early-childhood overweight and obesity

Mean (SD) weight of our study population was 8.35 (0.93) kg at 6 months, 10.08 (1.07) kg at 12 months, and 11.34 (1.15) kg at 18 months. Mean (SD) BMI of our participants was 17.97 (1.50) kg/m^2^ at 6 months, 17.43 (1.28) kg/m^2^ at 12 months, and 16.67 (1.19) kg/m^2^ at 18 months.

**Table 5** demonstrated the associations between cord blood metabolic factor levels and early-childhood overweight and obesity. After adjustments for maternal age, prepregnancy BMI, maternal education level, family income, parity, GDM, hypertensive disorders in pregnancy, ICP, child sex and birth weight, every mmol/L increase in cord blood TG concentration was significantly associated with decreased risks of overweight and obesity at 6 months [adjusted odds ratio (AOR) = 0.49; 95% CI = 0.29, 0.85; P = 0.011], 12 months (AOR = 0.58; 95% CI = 0.35, 0.96; P = 0.034), and 18 months (AOR = 0.46; 95% CI = 0.26, 0.83; P = 0.010). Increases in cord plasma TG/TC ratios were remarkably associated with decreased risks of overweight and obesity at 6 months (AOR = 0.10; 95% CI = 0.03, 0.35; P < 0.001), 12 months (AOR = 0.08; 95% CI = 0.02, 0.29; P < 0.001), and 18 months (AOR = 0.21; 95% CI = 0.06, 0.72; P = 0.013). Furthermore, every unit elevation in HbA1c significantly reduced the risks for overweight and obesity at 6 months (AOR = 0.81; 95% CI = 0.67, 0.98; P = 0.033), 12 months (AOR = 0.81; 95% CI = 0.67, 0.99; P = 0.037), and 18 months (AOR = 0.74; 95% CI = 0.60, 0.91; P = 0.005). However, there were no remarkable associations between other cord blood metabolic factors and early-childhood overweight and obesity outcomes.

**Table 5:** Associations between cord blood metabolic factors and early-childhood overweight and obesity.

Cord blood metabolic factors	6 months		12 months		18 months	
	AOR (95% CI)	*P*	AOR (95% CI)	*P*	AOR (95% CI)	*P*
TC	0.90 (0.75, 1.08)	0.269	1.05 (0.89, 1.26)	0.556	0.94 (0.78, 1.13)	0.510
TG	0.49 (0.29, 0.85)	0.011	0.58 (0.35, 0.96)	0.034	0.46 (0.26, 0.83)	0.010
LDL-C	0.84 (0.61, 1.15)	0.272	1.19 (0.89, 1.59)	0.246	0.83 (0.60, 1.15)	0.262
HDL-C	1.05 (0.71, 1.55)	0.814	1.19 (0.80, 1.76)	0.398	1.31 (0.87, 1.96)	0.195
TG/TC	0.10 (0.03, 0.35)	0.000	0.08 (0.02, 0.29)	0.000	0.21 (0.06, 0.72)	0.013
HDL/LDL	1.17 (0.96, 1.41)	0.120	0.91 (0.74, 1.11)	0.341	1.12 (0.92, 1.37)	0.262
Insulin	1.00 (0.98, 1.02)	0.999	1.01 (0.99, 1.02)	0.391	1.00 (0.99, 1.02)	0.811
C-peptide	1.03 (0.64, 1.67)	0.899	1.23 (0.76, 1.99)	0.403	0.85 (0.51, 1.41)	0.533
HbA1c	0.81 (0.67, 0.98)	0.033	0.81 (0.67, 0.99)	0.037	0.74 (0.60, 0.91)	0.005

Forward stepwise logistic regression analysis was applied to explore the relationship between cord blood metabolic factors and early-childhood overweight and obesity. Offspring overweight and obesity was defined as a BMI ≥85 percentile among the same age and same gender children. Model was adjusted for maternal age, prepregnancy body mass index, maternal education level, family income, parity, gestational diabetes mellitus, hypertensive disorders in pregnancy, intrahepatic cholestasis of pregnancy, child sex and birth weight.

AOR, adjusted odds ratio; CI, confidence interval; TC, total cholesterol; TG, triglyceride; LDL-C, low-density lipoprotein-cholesterol; HDL-C, high-density lipoprotein-cholesterol; HbA1c, glycosylated hemoglobin type A1C.

The optimal cutoff point proposed by ROC curve analysis for cord blood TG in predicting 18-month overweight and obesity was <0.29 mmol/L (sensitivity = 56.1%, specificity = 50.9%, AUC = 0.54). The optimal cutoff point for cord blood TG/TC in predicting 18-month overweight and obesity was <0.20 (sensitivity = 39.0%, specificity = 69.3%, AUC = 0.54). The optimal cutoff point for cord blood HbA1c in predicting 18-month overweight and obesity was <3.25% (sensitivity = 20.4%, specificity = 86.9%, AUC = 0.53).

## Discussion

The present prospective cohort study comprehensively displayed cord blood lipid and glucose metabolism in Chinese neonates and explored the associations between cord blood metabolic factors and growth indicators and overweight and obesity at the early stage of life. Previous research mostly investigated the associations between cord serum lipid profile, glucose metabolism and fetal growths and birth weights (6–10, 12). However, few studies followed up postnatal growth indicators and explored the associations between cord blood metabolic factors and early-childhood overweight and adiposity. Therefore, our study provided more epidemiological data of cord blood metabolic factors, improved insights into the associations between intrauterine metabolic environment and early-childhood growth, and indicated the underlying cord blood biomarkers for preventing childhood overweight and adiposity.

One outstanding finding from this study was that cord blood TG levels and TG/TC ratios were negatively associated with early-childhood growth. It has been reported that umbilical cord blood TG levels were negatively associated with fetal weights and birth weights (6–9). Our results further discovered inverse correlations between cord serum TG concentrations, TG/TC ratios and weight Z-scores and BMI Z-scores up to 18 months. These outcomes were in accordance with research work from Ye et al. (19) and Cao et al. (20). It has been shown that hypertriglyceridemia was closely related to islet β-cell dysfunctions, including interfered β-cell maturation, suppressed β-cell proliferation, inhibited insulin secretion, and induced insulin resistance. These disturbances hindered the activation of amino acid transport system, inhibited protein and fat synthesis, promoted lipolysis, and finally affected fetal growth and development, resulting in intrauterine growth restriction (21–24). At the same time, fetuses redistributed their nutrients for crucial organs (e.g., brain) to adapt to the adverse intrauterine environment, which further led to decreased fetal growth rate and birth weight (25). On the basis of the DOHaD theory, we speculated that fetal programming continued to play an important role in the growth patterns during the period of early childhood. Population-based cohort studies confirmed that fetal growth at different gestational periods was significantly associated with risks of impaired childhood growth, stunting, and obesity (26, 27). Increased fetal growth at each period was negatively associated with stunting and underweight at the age of 3 years (26). Conversely, intrauterine growth restriction infants were more prone to developing stunting when they were followed up to 18 months (27). These research findings supported metabolic disturbances and fetal growth restriction led to a long-term adverse effect on childhood growth. Thus, our findings provided convincing evidence for previous studies and figured out subsequent linear correlations sustained to early life.

Another interesting discovery from our research was that cord blood C-peptide and HbA1c levels had inverse correlations with early-childhood growth. This was a novel contribution rarely referred in previous studies. Once, a prior study discovered that cord serum C-peptide concentrations were positively associated with the incidence of neonatal macrosomia (28). An offspring follow-up research found that cord blood insulin concentrations were negatively associated with weight gain, skinfold thickness, and body fat percentage at early childhood (13, 14). Nevertheless, in another prospective cohort study, cord blood insulin concentrations were positively associated with skinfold thickness at age 1 year (15). Our study found weak associations between cord blood insulin concentrations and weight Z-scores, BMI Z-scores, and weight gains in infancy without clinical significance. These conflicting consequences suggested there still existed controversies, which deserved further explorations. It has been reported that umbilical blood insulin, C-peptide, and HbA1c were essential indicators reflecting glucose metabolic status of fetuses. Increased insulin, C-peptide, and HbA1c usually implied islet β-cell dysfunctions and insulin resistance, which was closely linked with dyslipidemia and amino acid transportation disorders (22, 29, 30). Fetal growth restriction and failure to thrive in early-childhood might occur as a result of impaired protein and lipid synthesis, transportation, and degradation (21–25). Furthermore, evidence has shown that oxidative stress and overproduction of reactive oxygen species (ROS) were involved in the process of metabolic dysfunction and obesity (31, 32). Thus, we speculated that multiple complicated factors contributed to the inverse associations between cord plasma C-peptide and HbA1c levels and weight Z-scores and BMI Z-scores at early childhood.

Compared with existing findings, there was an inconsistent result in our study. It has been demonstrated that cord plasma TG levels were positively associated with adiposity indicators among SGA infants but tended to be negatively associated with adiposity indicators among LGA infants in the research of Ye et al. (19). However, our outcomes did not find such opposite associations between TG levels and weight Z-scores or BMI Z-scores stratified by weight for gestational age, even after adjusting for confounding variables. From our perspective, the contradictory results might be explained by several factors affecting early-life growth including feeding pattern, dietary nutrition intake, physical activity, and other interference factors (especially catch-up growth in SGA infants). Future similar prospective studies were required to further explore the association and clarify the discrepancy.

Our study advanced insights regarding the relationship between cord blood metabolic factors and early-childhood overweight and obesity. Our consequences indicated that elevated cord plasma TG, HbA1c, and TG/TC ratios were associated with decreased risks for overweight and obesity. The results were consistent with findings from Cao et al. (20), which discovered that 22 triacylglycerols and diacylglycerols were negatively associated with early-onset overweight and obesity. A meta-analysis reviewed 22 studies and concluded that umbilical cord blood leptin was inversely associated with adiposity up to 3 years of age (16). In addition, two recent studies provided evidence convincing the involvement of cord blood metabolites (including cholestenone, branched-chain amino acid, and other metabolites) in the tendency to rapid postnatal growth and of rapid postnatal growth in the tendency to childhood overweight (33, 34). So far, explorations concerning the relationships between cord blood metabolites and childhood overweight and adiposity were very limited in past research. We contributed to finding the underlying cord blood biomarkers for predicting early-childhood overweight and obesity.

There were some limitations that could not be ignored in our study. Firstly, our study population was mainly enrolled from hospitals in Zhejiang Province. Given the huge population gross of China, a multicenter birth cohort study with a larger sample size and multiple nationalities would be more representative. Secondly, anthropometric measurements were obtained from different instruments by different clinicians and nurses. We could not rule out the possibility of measuring error. Thirdly, some maternal data (e.g., prepregnancy weight, height, and family income) were collected through self-reported questionnaires, which might be subject to recall bias. Fourthly, our participants were followed up to 18 months with weights and lengths measured at different time points. The study could be more ideal if other adiposity indicators such as subscapular and triceps skinfold thickness were included and longer periods were followed up. Finally, as mentioned before, childhood growth was impacted by several complicated factors (e.g., feeding pattern, dietary nutrition intake, physical activity, vitamin D supplement, etc.). Additional studies with confounding variables well-controlled are needed to elucidate the relationship between cord blood metabolic factors and growth and development in the future.

## Conclusion

Cord blood TG concentrations and TG/TC ratios were negatively associated with weight Z-scores and BMI Z-scores from birth to 18 months. Cord blood C-peptide and HbA1c levels had inverse correlations with early-childhood weight Z-scores and BMI Z-scores. Cord blood TG, TG/TC ratios, C-peptide, and HbA1c had negative correlations with weight gains from birth to 6 months, but the correlations attenuated as time went on. Increased cord plasma TG, HbA1c levels, and TG/TC ratios were associated with decreased risks for overweight and obesity at 6 months, 12 months, and 18 months. Therefore, cord blood metabolic factors were significantly associated with early-childhood growth patterns.

## Data availability statement

The raw data supporting the conclusions of this article will be made available by the authors, without undue reservation.

## Ethics statement

The studies involving human participants were reviewed and approved by Clinical Research Ethics Committee of Yongkang, Huzhou, Cixi, Ninghai, Shaoxing, Yiwu, Dongyang and Wenling Maternal and Child Health Care Hospitals. Written informed consent to participate in this study was provided by the participants’ legal guardian/next of kin.

## Author contributions

W-YJ participated in study design, data acquisition, literature review, statistical analysis and drafted the manuscript. X-YC, TH, YJ, T-TC and Z-HW contributed to data acquisition, literature review and performed statistical analysis. Z-WZ and Z-YZ conceived the study, participated in its design and were the corresponding authors of this manuscript. All the authors read and approved the final submitted version.
